# Comparison of family health history to personal genomic screening for risk assessment of colon cancer

**DOI:** 10.1186/1897-4287-9-S1-O2

**Published:** 2011-03-10

**Authors:** Brandie Heald, Charis Eng

**Affiliations:** 1 Genomic Medicine Institute, Cleveland Clinic, Cleveland, OH 44195, USA

## Background

Family history-based risk assessment (FHRA) is a validated genetic tool for identifying those at moderate and high risks of disease. The latter usually result from gene mutations conferring high risks of disease. Genome-wide association studies (GWAS) have resulted in single nucleotide polymorphisms (SNP) statistically associated with low- to moderate-level risks of diseases or traits. With increased access and decreasing price-point for SNP profile-based risk assessment for common diseases, such as colon cancer, health-professionals and consumers are questioning how this type of genetic risk assessment compares to the standard of FHRA. To date, there has been limited study of concordance for these two methods of disease-risk assessment.

## Materials and methods

We compared FHRA with Navigenics personal genomic screening (PGS) for colon cancer in 44 subjects (22 males, 22 females) ascertained from our genetics clinics at the Genomic Medicine Institute at Cleveland Clinic. Each subject was categorized as low/general population, moderate, or high risk based on FHRA and PGS results. Concordance of FHRA and PGS categorizations was assessed with the kappa (K) statistic. We also assessed each subject’s hereditary risk based on clinical criteria and/or validated gene test results.

## Results

Both FHRA and PGS placed 44% of participants into the same risk categories. Overall, however, the concordance between FHRA and PGS risk assessments was low (K=-0.05). There were 17 subjects with a moderate colorectal cancer risk on PGS whereas FHRA classified 12 as general population risk and 3 at high risk. Similarly, FHRA classed 7 subjects as high risk for colorectal cancer whereas none were noted as high risk by PGS. FHRA classified 9 subjects with hereditary risk for colorectal cancer, of whom 5 met syndromic clinical criteria while the remaining 4 had family histories meeting hereditary colorectal cancer criteria, but none of these 9 were classified by PGS as high risk (p=0.0001).

## Conclusions

FHRA and PGS may be complementary tools for colon cancer risk assessment. However, evaluation of family history remains the gold standard, and this should be used to clinically evaluate an individual’s risk of developing colon cancer until further research demonstrates PGS can be integrated with family-based assessment to increase sensitivity.

